# Supplementary material for: The interaction of transcription factors controls the spatial layout of plant aerial stem cell niches
Source: NPJ Syst Biol Appl. 2018 Sep 6;4:36. doi: 10.1038/s41540-018-0072-1 (PMC6127332; doi:10.1038/s41540-018-0072-1)
Supplement: Supplementary file 1 — Supplementary Figures [file 41540_2018_72_MOESM1_ESM.pdf]

Supplementary information

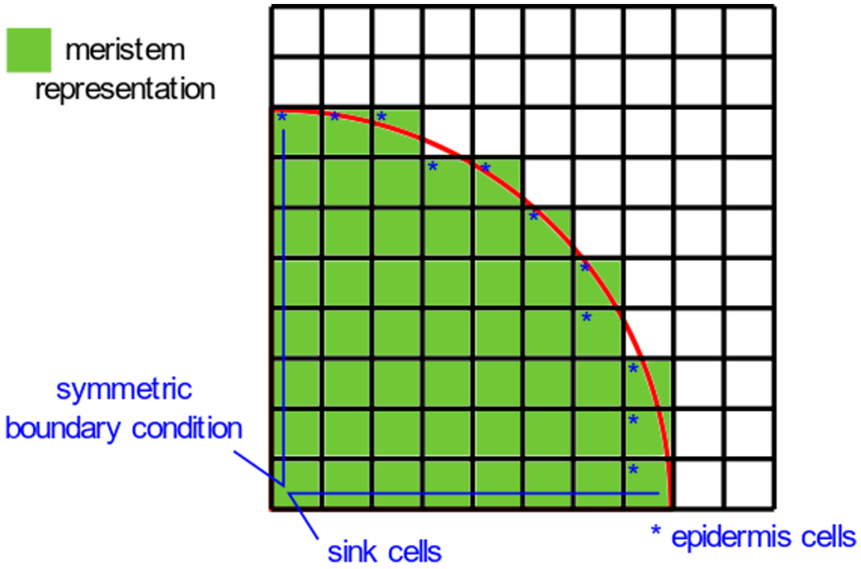

Figure S1: **Meristem geometry layout.**

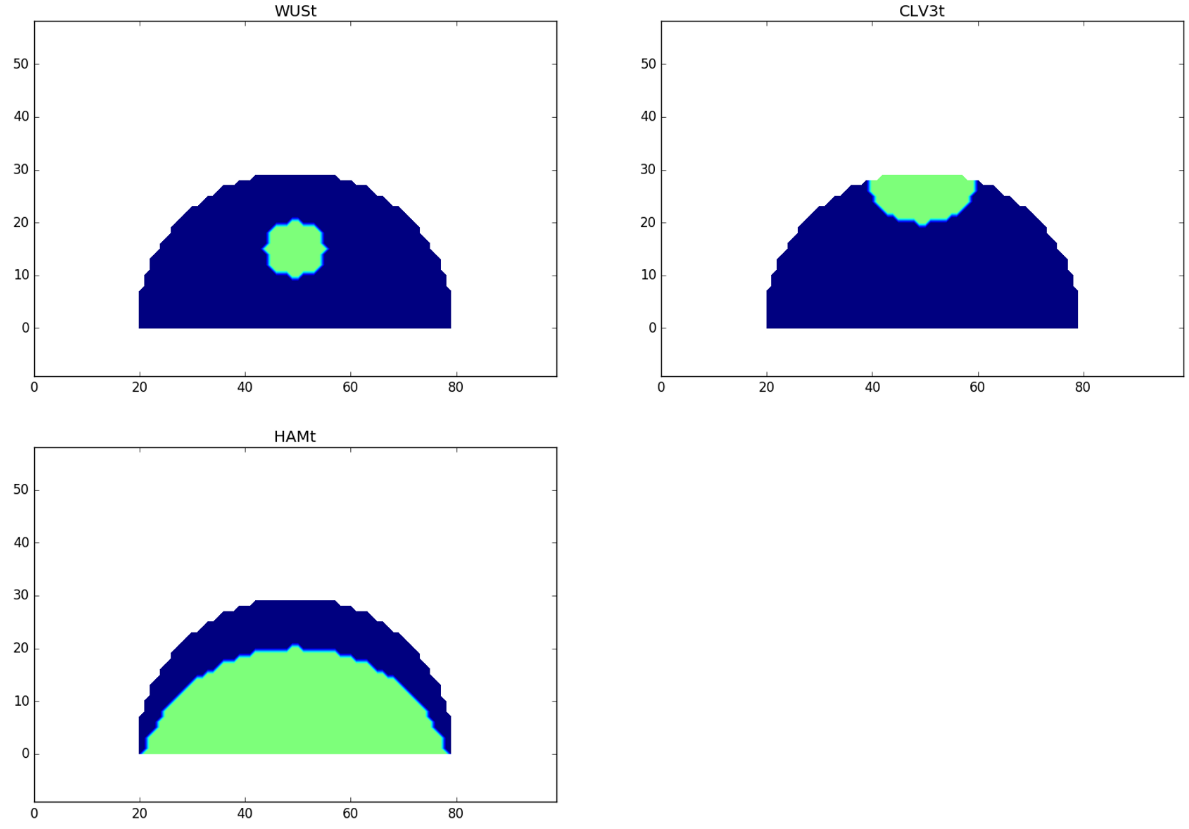

Figure S2: **Optimisation target domains for *WUS*, *CLV3* and *HAM*.**

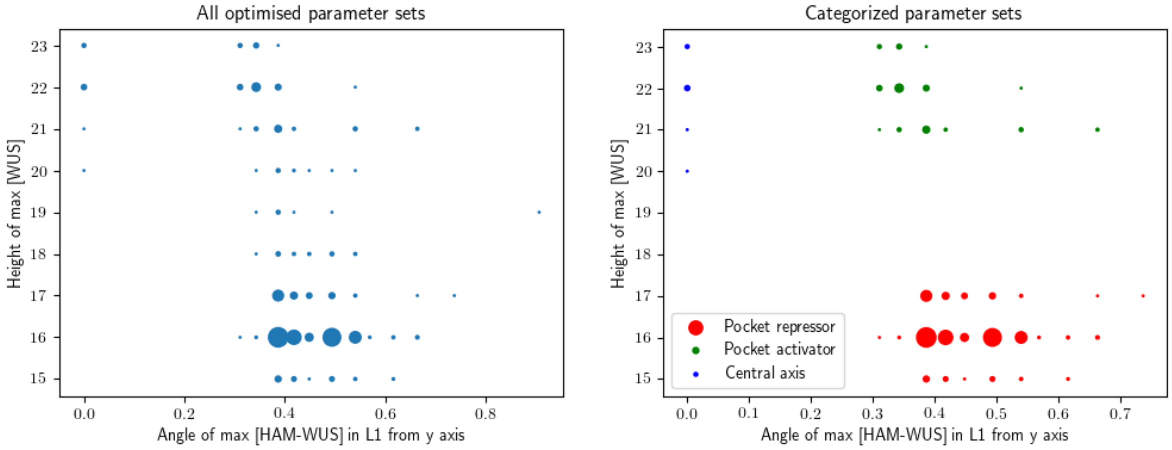

Figure S3: **Parameter sets categorization.** In both panels, the x-axis represents the angle from the meristem y-axis at which the maximal concentration of HAM-WUS dimers is found in the L1. The y-axis displays the height at which the maximal concentration of WUS monomers is found (along the meristem y-axis). The size of the points shows the amount of parameters sets found. The left panel shows all parameter sets obtained from optimisations and the right panel shows the categorized parameter sets.

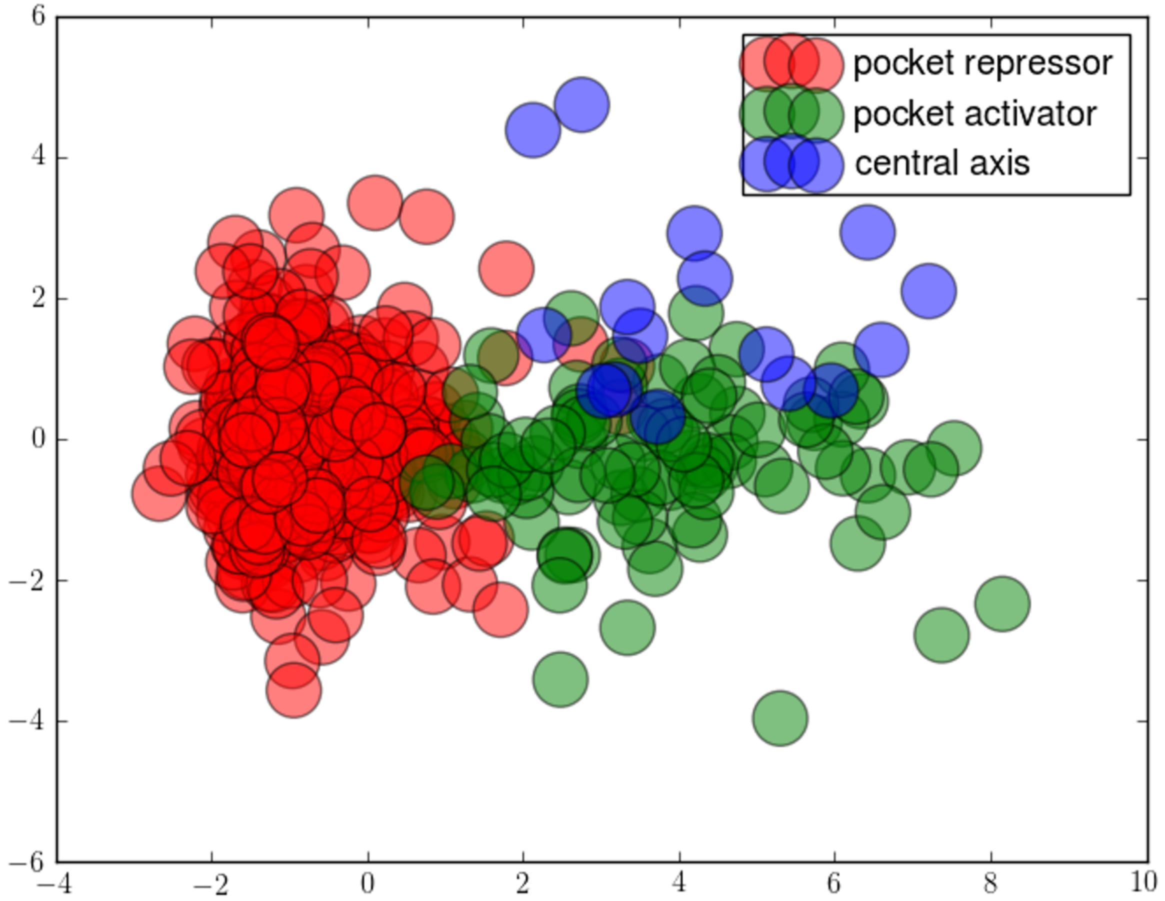

Figure S4: **The three categories of behaviour can be separated in the parameter space.** The base 10 logarithm of parameter values were centered and scaled before running a linear discriminant analysis. The figure displays the projection of the parameter sets along the most discriminative directions. The linear discriminant analysis algorithm is from the scikit-learn python package.

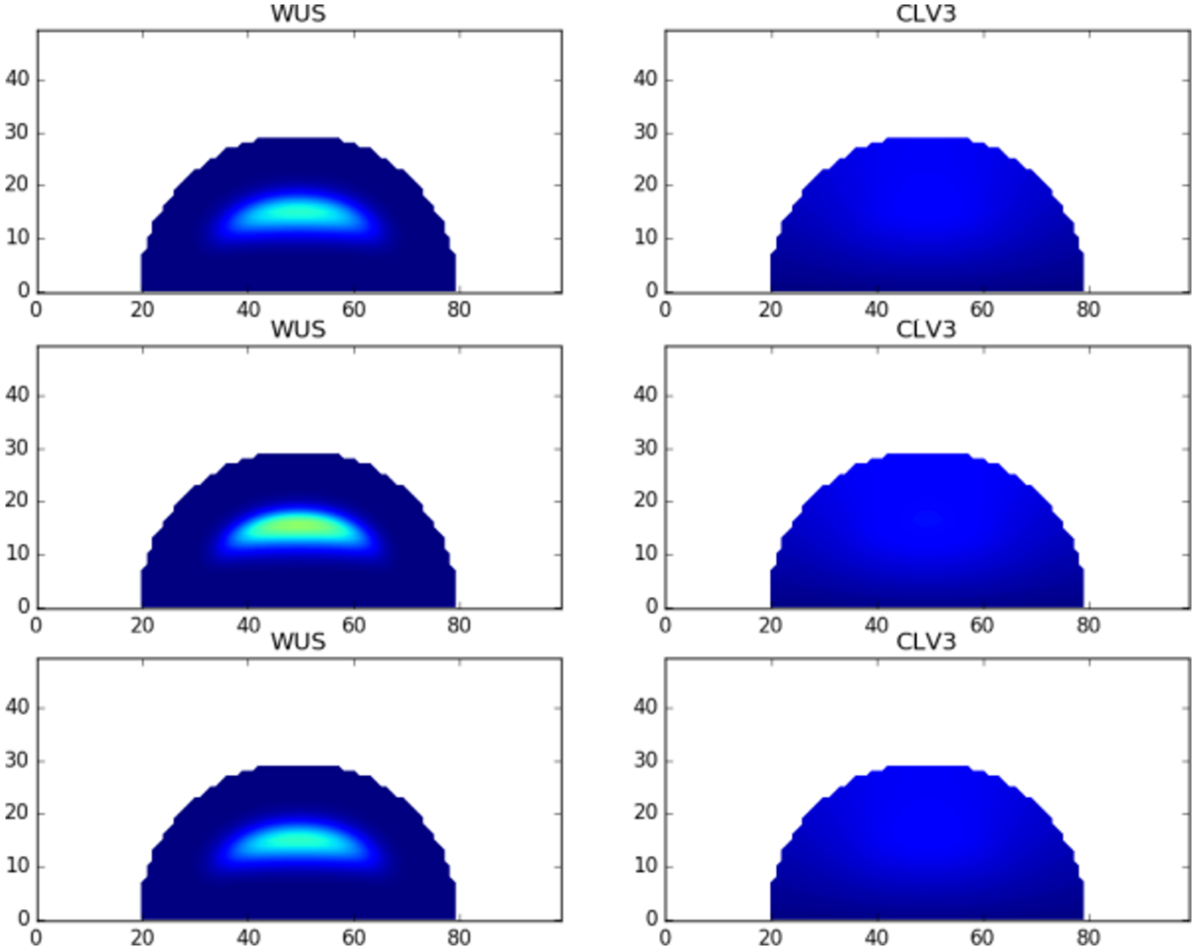

Figure S5: **Optimisation results for a model based entirely on WUS regulating *CLV3*.** The optimisation procedure was adapted to test a model where WUS is the only regulator of *CLV3* expression. The rows show three examples of the results obtained: in this scenario the best *CLV3* expression is a faint hue covering the *WUS* expression domain and the tip of the meristem. Expression of genes in the panels goes from blue (null) to red (twice the optimisation target expression and above) *via* green (optimisation target).

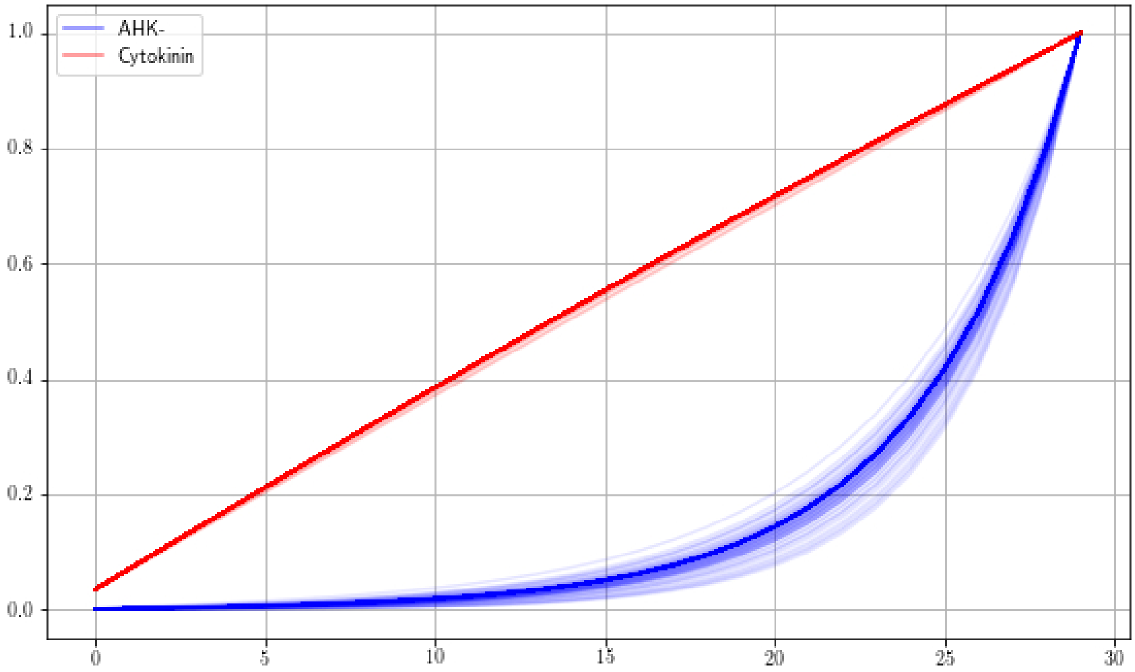

Figure S6: **Gradients of AHK repressor (blue) and cytokinin (red)** along the central axis of the meristem, for all optimised parameter sets. The apex is to the right and the stem is to the left and concentrations are normalised.

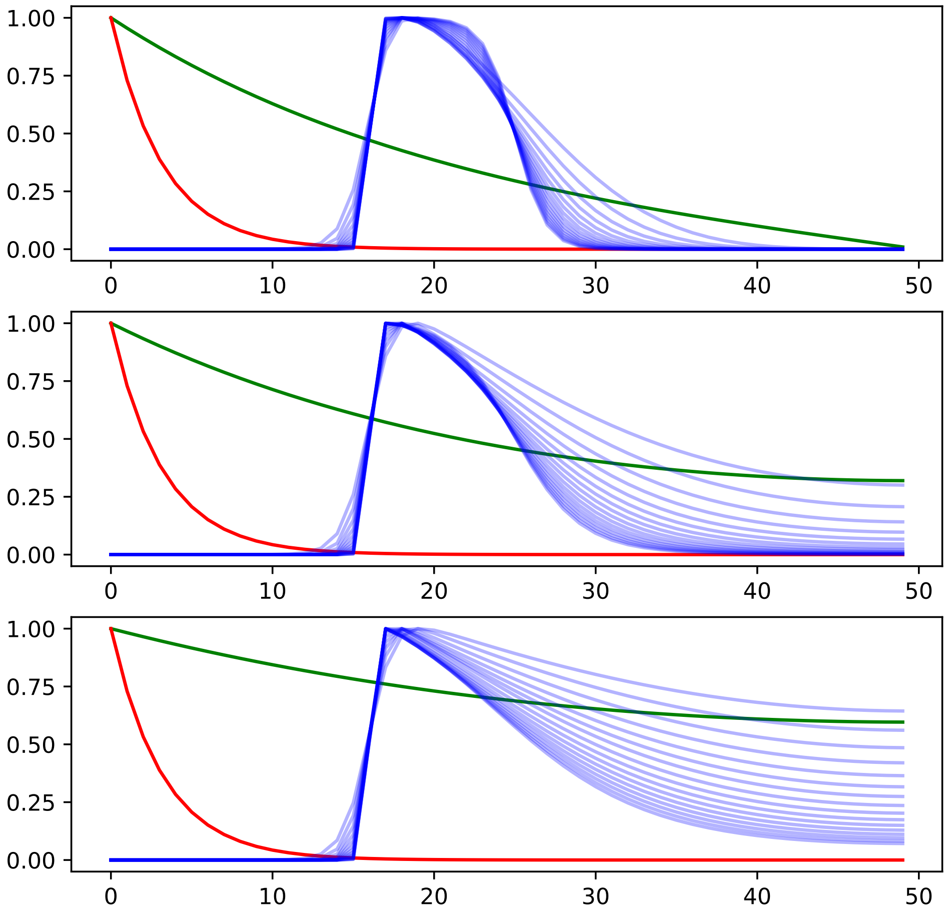

Figure S7: **Various cytokinin gradients can correctly pattern *WUS* expression.** We tested a 1D *WUS* expression model where cytokinin (green) acts as an activator and AHK repressor (red) acts as a repressor. In order to assess the importance of the cytokinin gradient, we tested three gradients ranging from sharp (top) to the shallow (bottom). The expression of *WUS* is described by Hill functions, with varying coefficients (ranging from 4 to 20); the higher the coefficients the sharper *WUS* expression.

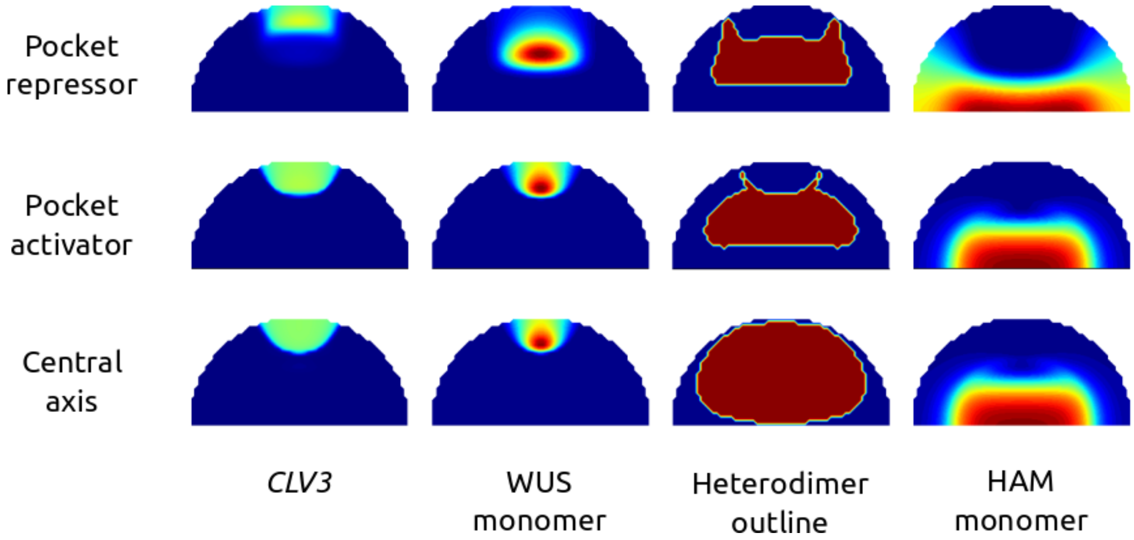

Figure S8: **A no flux boundary allows the patterning of the gene expression domains.** Rows display the three categories: pocket repressor, pocket activator and central axis. Columns display the expression of *CLV3*, WUS monomer and the outline of the concentration of the heterodimer (both used for categorization), and HAM monomer (the no flux boundary makes the concentration peak at the bottom of the tissue). The color scales are the same as Figure 1.
